# Molecular epidemiology of drug resistance and transmission of *Mycobacterium tuberculosis* in Meigu County, Sichuan Province, China

**DOI:** 10.1128/spectrum.02685-25

**Published:** 2025-12-29

**Authors:** Yang Deng, Xin He, Cise Bibu, Yi-Xuan Ren, Yong Fang, Labu Qubi, Ai-Zhen He, Wulang Acheng, Chuan-You Li, Jie Zhang, Jie Li

**Affiliations:** 1 Beijing Center for Disease Prevention and Control, Beijing, China; 2 School of Public Health, Capital Medical University 379397, Beijing, China; 3 Meigu County Disease Prevention and Control Center, Sichuan, China; University of Chicago, Chicago, Illinois, USA

**Keywords:** *Mycobacterium tuberculosis*, drug resistance, whole-genome sequencing, transmission clusters, molecular epidemiology, Meigu County

## Abstract

Tuberculosis (TB) remains a major public health challenge in China. Meigu County, in Liangshan Yi Autonomous Prefecture, Sichuan Province, is severely affected by a high TB burden, a situation exacerbated by its geographic isolation and socioeconomic constraints. This study used DNA microarray technology to assess drug resistance in 378 *Mycobacterium tuberculosis* (*M. tuberculosis*) isolates from bronchoalveolar lavage fluid samples collected in Meigu County from 2022 to 2024. During this period, the drug resistance rates of rifampicin, isoniazid, and multidrug-resistant TB initially rose and then declined. Only the variation in isoniazid resistance reached statistical significance (*χ*² = 6.462, *P* = 0.038). Age-specific analysis revealed a significantly higher prevalence of isoniazid resistance among individuals aged 19–60 years (*χ*² = 7.034, *P* = 0.022). Whole-genome sequencing was further applied to 123 isolates collected from the same geographical area and sample type in 2024. Genomic analysis detected drug-resistant mutations in 8.9% (11/123) of the isolates, with dominant mutations including *rpoB* p.Ser450Leu and *katG* p.Ser315Thr. Lineage 2 and Lineage 4 strains demonstrated comparable prevalence, with no statistically significant difference observed among the cases analyzed in 2024. Phylogenetic clustering based on a ≤12 single-nucleotide polymorphism (SNP) threshold grouped 31.7% (39/123) of the strains into 15 distinct transmission clusters, reflecting ongoing community spread. Clustering was significantly associated with amikacin resistance (*P* = 0.001). Discrepancies in resistance predictions between bioinformatic tools highlighted the necessity for standardized genomic surveillance protocols. These results offer the first comprehensive genomic characterization of *M. tuberculosis* transmission and resistance in Meigu County, underscoring the critical need for targeted public health strategies to interrupt transmission in this high-incidence setting.

**IMPORTANCE:**

This study provided a critically important investigation into the molecular epidemiology of drug-resistant tuberculosis in Meigu County, a remote and high-burden region in Sichuan Province, China. By combining DNA microarray chip technology and whole-genome sequencing, the researchers characterized the genetic makeup, drug resistance patterns, and transmission dynamics of strains of *Mycobacterium tuberculosis* circulating in this underserved community. They identified specific mutations responsible for resistance to key anti-tuberculosis drugs and revealed ongoing local transmission through genomic clustering analysis. The findings highlighted the urgent need for improved public health interventions, standardized treatment protocols, and enhanced genomic surveillance in regions with limited healthcare resources. This work offered the comprehensive genomic insight into tuberculosis transmission in Meigu County and served as a model for understanding and combating drug-resistant tuberculosis in other high-incidence, economically disadvantaged areas worldwide.

## INTRODUCTION

Tuberculosis (TB), caused by *Mycobacterium tuberculosis*, remains a leading infectious cause of mortality worldwide. The recent resurgence in global TB incidence, with over 10 million new cases reported annually since 2021, underscores its persistent threat. The timely diagnosis of active TB is crucial for initiating appropriate treatment and controlling transmission. Conventional methods like smear microscopy and culture are limited by suboptimal sensitivity and potential contamination, particularly in paucibacillary or critically ill patients (1–3). These challenges are exacerbated when patients cannot produce adequate sputum samples. Bronchoalveolar lavage fluid (BALF) has been widely utilized as a preferred diagnostic specimen for respiratory tract infections. This sampling technique offers distinct advantages over expectorated sputum by obtaining representative specimens from the lower respiratory tract while minimizing upper airway contamination. Currently, BALF has been widely applied in multiple aspects of TB diagnosis. For instance, BALF samples are utilized for metagenomic next-generation sequencing to analyze the pulmonary microbiome, as well as for Xpert MTB/RIF assays to assist in the diagnosis of pulmonary TB (4, 5).

China continues to bear a significant proportion of the global TB burden. A particularly concerning development is the growing challenge of drug-resistant TB (DR-TB), notably rifampicin (RFP)-resistant TB (RR-TB) and multidrug-resistant TB (MDR-TB), the latter defined as resistance to at least both RFP and isoniazid (INH). These strains severely compromise treatment outcomes and pose major public health risks. In 2023, China accounted for 7.3% of global MDR/RR-TB cases (6), necessitating a deeper understanding of their transmission patterns to inform effective control strategies.

While traditional molecular epidemiology has provided initial insights into TB transmission, conventional genotyping methods suffer from limited discriminatory power and reliability in delineating recent transmission chains (7). The advent of whole-genome sequencing (WGS) has revolutionized the field, offering unprecedented resolution for reconstructing transmission networks and identifying resistance mutations, even in the absence of complete epidemiological data (8, 9).

Despite being one of China’s most TB-endemic regions, Sichuan Province—situated in the country’s western interior—has made less progress in curbing the disease burden than its eastern coastal counterparts. Meigu County (102°53′E to 103°21′E, 28°02′N to 28°54′N) in Sichuan’s Liangshan Yi Autonomous Prefecture is a predominantly rural region with 242,000 residents (81.82% rural population), where 97.1% identify as Yi ethnicity. The county faces a severe TB epidemic, with 2019–2023 surveillance data showing an alarming average annual incidence of 375.23/100,000—nine times China’s 2023 national rate (43.49/100,000) (10), highlighting critical gaps in TB control. The reported incidence rates in this county rose significantly in 2022 and 2023, reaching 481.48 per 100,000 and 453.73 per 100,000 respectively, which were higher than the incidence rates of most of the 30 countries with high TB burdens worldwide. In response, the Chinese government has prioritized TB control in such underdeveloped and remote areas through specialized policies, financial support, and enhanced resources (11). As part of these enhanced control measures, local health authorities in Meigu have formally approved BALF as a standard specimen for all suspected pulmonary TB patients to strengthen diagnostic capacity.

Mounting evidence implicates MDR-TB strain transmission as a key driver of China’s persistent epidemic. Although WGS has proven invaluable for elucidating transmission networks in developed eastern regions (12–15), this high-prevalence area remains understudied at the population genomic level, creating critical gaps in our knowledge of local transmission epidemiology. To address this, we first performed drug resistance profiling using DNA microarray on isolates of *M. tuberculosis* from BALF samples of patients in Meigu County (2022–2024). We then conducted a retrospective WGS-based study on isolates from 2024 to characterize drug resistance patterns, genetic diversity, transmission dynamics, and associated risk factors, providing critical evidence for guiding targeted interventions against MDR/RR-TB in this underserved region.

## MATERIALS AND METHODS

### Study population and sample collection

Situated in the mountainous terrain of southwest China, Meigu County constitutes an integral part of Sichuan Province’s Liangshan Yi Autonomous Prefecture. Our study cohort consisted of all culture-confirmed pulmonary TB cases diagnosed at Meigu County Hospital during the surveillance period from 2022 to 2024. BALF samples were collected from each patient with culture-positive TB, yielding a total of 378 BALF samples. Written informed consent was obtained from each patient or their family, and BALF samples were extracted following standard operational procedures. The demographic characteristics of the patients were analyzed and are presented in the Results section.

### Detection of DR genes of isolates using DNA microarray chip method

For each specimen collected between 2022 and 2024, 2–3 mL of BALF sample was processed with an equal volume of freshly prepared 4% NaOH solution (1:1–2 vol/vol). The mixture was vortexed until homogeneous and incubated at room temperature for 15–20 minutes for digestion. After digestion, 1 mL of the liquefied supernatant was centrifuged at 5,000 rpm for 5 minutes. The pellet was washed with 1 mL of sterile 0.9% physiological saline and centrifuged again under identical conditions to remove residual NaOH. An aliquot of 0.1 mL of the resulting specimen was inoculated into two tubes of acidified Löwenstein-Jensen (L-J) medium and incubated at 37°C for weeks 2–3. Finally, a total of 378 culture-positive isolates of *M. tuberculosis* were obtained.

DNA purification was performed using the Extractor 36 Rapid Nucleic Acid Extraction System. Single colonies from L-J solid medium were suspended in lysis buffer and processed through sequential steps of vortex mixing (5 minutes), heat treatment (95°C for 5 minutes), and centrifugation (5,000 rpm for 1 minute). The resulting DNA extract was used directly for subsequent PCR amplification. The purified DNA was analyzed using the CapitalBio *M. tuberculosis* Drug Resistance Gene Detection Kit (Beijing, China) according to the manufacturer’s protocol. The workflow included multiplex PCR amplification, microarray hybridization at 50°C for 120 minutes, and chip scanning using a LuxScan 10K-B microarray scanner. Results were interpreted against the kit’s reference database with a ≥95% confidence threshold. For participants with multiple drug susceptibility test results, the most resistant profile was selected for final analysis.

### DNA extraction, WGS, and bioinformatic analysis

For each patient enrolled in 2024, isolates were cultured from BALF samples, and genomic DNA was subsequently extracted from the bacterial cultures. The extracted DNA was processed into a sequencing library using the cetyltrimethylammonium bromide method. DNA libraries were prepared using the FS DNA Lib Prep Kit V6 (RK20259, Illumina) and sequenced in 150 bp paired-end mode on an Illumina NovaSeq 6000 platform (Illumina, San Diego, CA, USA). The raw sequencing reads underwent comprehensive bioinformatic processing. *De novo* assembly was performed using SOAPdenovo (v1.05), followed by alignment to the *M. tuberculosis* H37Rv reference genome (GenBank: GCA_000195955.2). Variant calling was conducted with Freebayes (v1.0.13), and subsequent variant annotation was carried out using SnpEff (v5.2f) to characterize mutation effects. Finally, genome completeness and contamination values were estimated using the CheckM pipeline (16).

Each isolate underwent variant detection and annotation, with WGS data cross-referenced against established databases of drug resistance-associated mutations. Antimicrobial resistance profiles were computationally predicted using TB-Profiler v6.6.5 (https://github.com/jodyphelan/TBProfiler) (17) and Mykrobe v0.10.0 (https://www.mykrobe.com) (18), leveraging the TB Drug Resistance Database (https://github.com/jodyphelan/tbdb) to analyze known resistance markers. Variant analysis was performed with Snippy v4.6.0 to generate a SNP matrix, followed by clustering (threshold: <12 SNPs; snp-dists v0.7.0) and maximum-likelihood phylogenetic reconstruction (FastTree v2.2), visualized via iTOL (https://itol.embl.de/).

### Statistical analysis

Statistical analyses were performed using SPSS software (version 26, SPSS Inc., Chicago, IL, USA). Categorical variables were compared using *χ* tests or Fisher’s exact tests, as appropriate. Risk factors associated with transmission clusters were identified through univariate and multivariate logistic regression analyses, with results expressed as odds ratios (ORs) and 95% confidence intervals (*CIs*). A *P*-value of <0.05 was considered statistically significant.

## RESULTS

### Demographic characteristics of pulmonary TB patients

**Fig 1:**
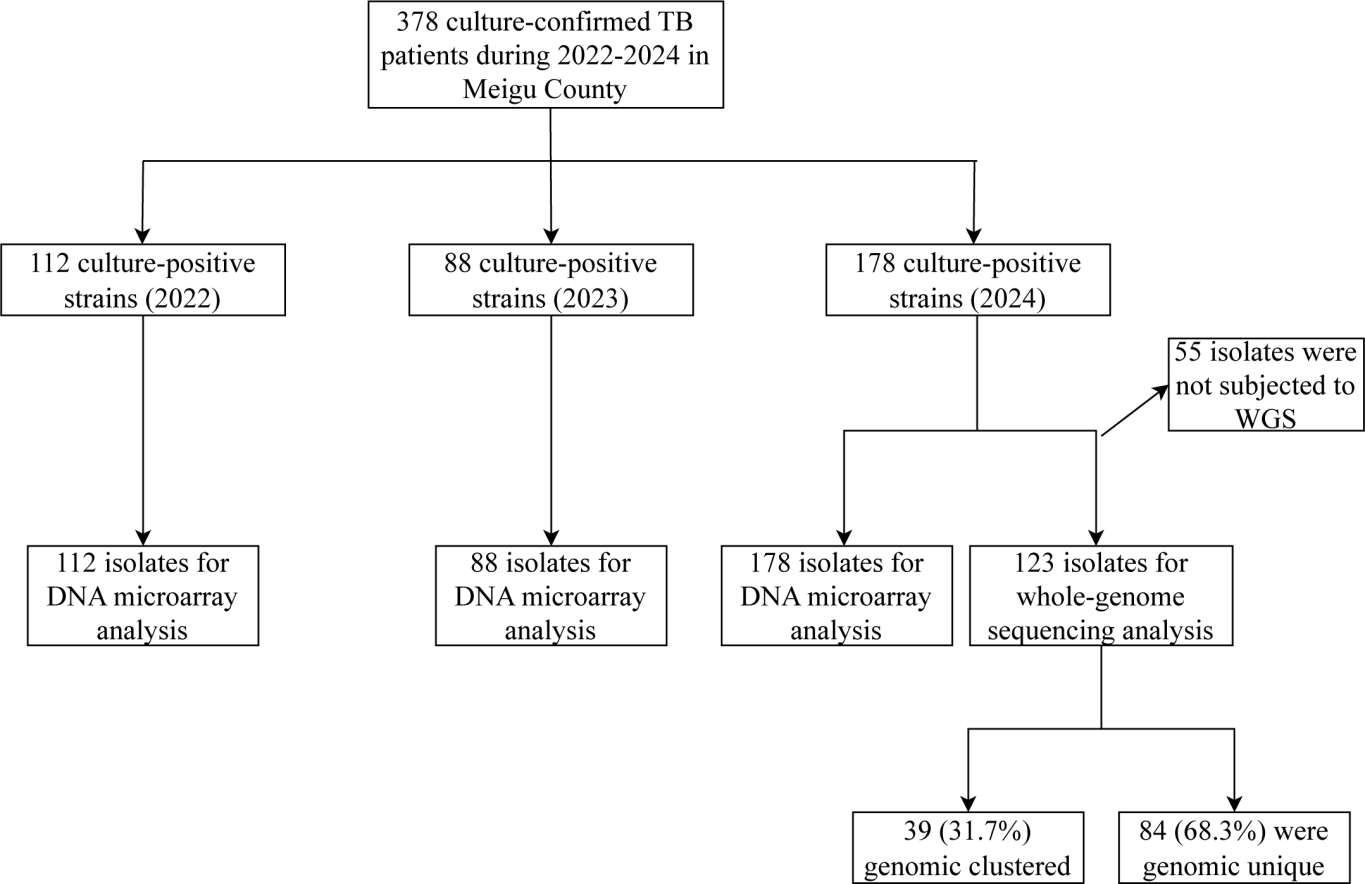
Patient inclusion flowchart.

Active surveillance conducted throughout 2022–2024 in Meigu County identified 378 bacteriologically confirmed pulmonary TB cases through the local Tuberculosis Surveillance System. The case series comprised exclusively Yi ethnic population, reflecting the demographic composition of this endemic region. All cases were laboratory-confirmed through standard bacteriological diagnostic methods, ensuring case verification accuracy. Molecular analysis of 378 culture-positive bronchoalveolar lavage (BAL) specimens confirmed *M. tuberculosis* complex infection in all cases, forming the basis of our study cohort (Fig. 1).

### Drug resistance profiling of isolates by DNA microarray chip method

The study population demonstrated a male predominance, with 237 cases (62.7%), compared to 141 female patients (37.3%). No statistically significant gender-based differences were observed in the prevalence of RR-TB, INH-resistant TB (HR-TB), or MDR-TB (all *P*-values > 0.05; Table 1). The age distribution spanned a wide range from pediatric cases (3 years old) to elderly patients (88 years old), with a median age of 35 years. Age distribution revealed 48 pediatric cases (3–18 years), 277 adult cases (19–60 years), and 53 elderly cases (≥61 years). The 19–60 age group exhibited higher proportions of resistant cases among the total clinical isolates analyzed in our study cohort, with particularly notable differences in INH-resistant strains (*P* < 0.05; Table 1). It should be noted, however, that this finding is based on the distribution of clinical isolates collected and may be influenced by the underlying age structure of the population in Meigu County. Temporal analysis from 2022 to 2024 indicated a consistent pattern of initial increase followed by subsequent decline in resistance rates for RR-TB, HR-TB, and MDR-TB. However, only INH resistance showed statistically significant fluctuations over time (*χ*² = 6.462, *P* = 0.038), as detailed in Table 1.

**TABLE 1:** Multivariate analysis of factors influencing drug resistance patterns in clinical isolates of *M. tuberculosis^a^*

Factor	RR-TB				HR-TB				MDR-TB			
	No.	%	*χ*²	*P*	No.	%	*χ*²	*P*	No.	%	*χ*²	*P*
Year												
2022 (*n* = 112)	7	6.25	1.058	0.596	7	6.25	6.462	**0.038^*b*^**	3	2.68	1.978	0.384
2023 (*n* = 88)	9	10.22			8	9.09			4	4.55		
2024 (*n* = 178)	15	8.43			4	2.25			3	1.69		
Gender												
Male (*n* = 237)	19	8.02	0.029	0.504	13	5.49	0.28	0.395	4	1.69	2.263	0.122
Female (*n* = 141)	12	8.51			6	4.26			6	4.26		
Age (years)												
<18 (*n* = 48)	1	2.08	4.676	0.093	0	0	7.034	**0.022**	0	0	2.418	0.261
18–60 (*n* = 277)	28	10.11			19	6.86			10	3.61		
>60 (*n* = 53)	2	3.77			0	0			0	0		

^*a*^ RR-TB, RFP-resistant TB; HR-TB, INH-resistant TB; MDR-TB, multidrug-resistant TB.

^*b*^ Bold values indicates the *P*-value is less than 0.05, highlighting the comparisons where the inter-group differences achieve statistical significance.

### Detection of drug resistance-associated mutations by DNA microarray chip method

Genotypic analysis of 31 RR *M. tuberculosis* clinical isolates through DNA microarray revealed distinct mutation patterns in the *rpoB* gene. The majority of strains (80.6%, *n* = 25) exhibited single-nucleotide polymorphisms (SNPs), while a significant minority (19.4%, *n* = 6) demonstrated double mutations in this critical drug resistance-determining region. The predominant mutations occurred at *rpoB* codon 531 (C->T; 32.3%, 10/31), codon 511 (T->C; 16.1%, 5/31), and codon 516 (A->T/A->G; 10.5%, 5/31), while less frequent variants included codon 526 (A->T/C->G; 6.5%, 2/31), codon 533 (T->C; 6.5%, 2/31), and codon 513 (A->C; 3.2%, 1/31). Notably, six strains (19.4%) displayed complex mutation profiles: four (12.9%) had concurrent *rpoB* 511 (T->C) and *rpoB* 516 (G->T/A->T/A->G) mutations, one (3.2%) possessed both *rpoB* 516 (A->T) and *rpoB* 526 (C->G), and one (3.2%) exhibited *rpoB* 511 (T->C) combined with *rpoB* 526 (A->T). Among INH-resistant strains, 15 (79.0%) carried the *katG* 315 (G->C) mutation, while 4 (21.1%) showed the *inhA* −15 (C->T) promoter mutation (complete mutation profiles are presented in Table 2).

**TABLE 2:** Distribution of drug resistance-associated mutations in isolates of *M. tuberculosis* in 2022–2024

Gene	Mutation site	Mutation type	No. of isolates	Mutation frequency (%)
*rpoB*	511	T->C	5	16.1
	513	A->C	1	3.2
	516	A->T; A->G	5	16.1
	526	A->T; C->G	2	6.5
	531	C->T	10	32.3
	533	T->C	2	6.5
	516, 526	516 (A->T); 526 (C->G)	1	3.2
	511, 516	511 (T->C); 516 (G->T;A->T;A->G)	4	12.9
	511, 526	511 (T->C); 526 (A->T)	1	3.2
*katG*	315	G->C	15	79.0
*inhA*	−15	C->T	4	21.1

### Drug resistance profile based on WGS

Among the culture-positive isolates obtained in 2024, 55 isolates were prioritized for drug resistance profiling using DNA microarray chip methods, which resulted in insufficient residual sample volume for subsequent WGS. Therefore, genomic analysis was performed on 123 BAL-derived *M. tuberculosis* isolates that had adequate sample volumes for both culture and WGS.

Quality assessment using CheckM confirmed high-quality genome assemblies for all sequenced isolates, meeting stringent thresholds (>95% completeness, <10% contamination). Genomic analysis revealed size variations (4.3–4.8 Mbp) and G + C content ranges (64.0%–65.6%) among the draft assemblies of these clinical isolates. Detailed assembly characteristics, including genome size, N50 length, and contig statistics, are presented in Table S1.

**Fig 2:**
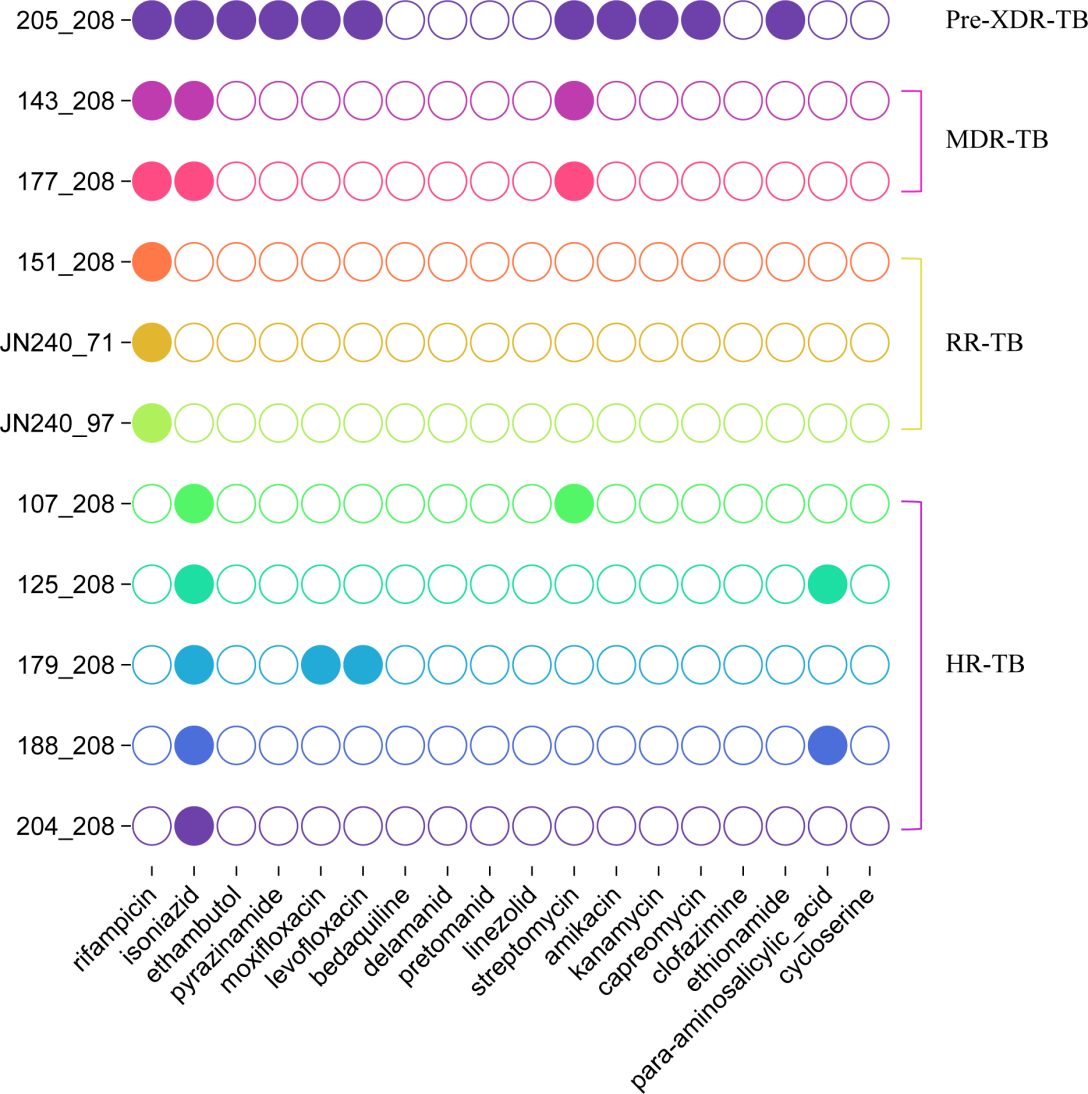
Drug resistance-associated mutations in 11 isolates of *M. tuberculosis* identified by TB-Profiler (WGS analysis). Pre-XDR-TB: it means the TB bacteria are resistant to the two most powerful first-line TB drugs (INH and RFP), which is called MDR-TB. Additionally, they are resistant to either a class of second-line drugs called fluoroquinolones (e.g., levofloxacin and moxifloxacin) or to at least one of the second-line injectable drugs (e.g., amikacin, kanamycin, and capreomycin).

Genetic variants of multiple genes associated with drug resistance were identified by WGS (Fig. 2). Among 123 clinical isolates, only 11 (8.9%) harbored resistance-associated mutations in their genomes. WGS revealed diverse resistance-associated mutations across the clinical isolates: for RFP, mutations in *rpoB* included p.Ser450Leu (*n* = 3), p.Leu452Pro (*n* = 1), p.Leu449Gln (*n* = 1), p.Asp435Gly (*n* = 1), and p.Met434Val (*n* = 1); INH resistance involved *katG* p.Ser315Thr (*n* = 4) and c.463delT (*n*=1) and *inhA* c.-777C>T (*n* = 1) and p.Ser94Ala (*n* = 1), plus *ahpC* promoter mutations (c.-74G>A and c.-81C>T; *n* = 1 each); fluoroquinolone resistance was derived from *gyrA* p.Asp94Gly (*n* = 2), conferring cross-resistance to both moxifloxacin and levofloxacin; and aminoglycoside resistance included streptomycin-associated *rpsL* p.Lys43Arg (*n* = 2), *gid* c.351delG (*n* = 1), and *rrs* n.514A>C (*n* = 1), while a shared *rrs* n.1401A>G mutation (*n* = 1) mediated cross-resistance to amikacin, kanamycin, and capreomycin. Additional mutations affected are as follows: ethambutol (*embB* p.Met306Ile; *n* = 1), pyrazinamide (*pncA* p.Gln141Pro; *n* = 1), ethionamide (*inhA* c.-777C>T/p.Ser94Ala; *n* = 1 each), and para-aminosalicylic acid (*thyX* c.-16C>T and *folC* p.Ile43Thr; *n* = 1 each).

Previous studies established that a C->T substitution at codon 531 of the *rpoB* gene confers the p.Ser450Leu amino acid change in the RpoB protein. Similarly, a T->C substitution at codon 533 resulted in the p.Leu452Pro alteration (19). Additionally, the most prevalent mutation in the *katG* gene is a point mutation at codon 315, where an AGC to ACC change results in the substitution of serine by threonine, denoted as p.Ser315Thr in the KatG protein (20). In this study, only four isolates (strains 151_208, 177_208, JN240_71, and 107_208) exhibited complete concordance between WGS resistance predictions by TB-Profiler and DNA microarray results, where all identified resistance mutations were consistently detected by both methods. WGS analysis via TB-Profiler identified an isoniazid resistance-associated mutation (*ahpC* c.-81C->T) in strain 204_208, while DNA microarray detected a RFP resistance mutation (*rpoB* 531 [C->T]) (Table 3). Inter-tool comparison demonstrated variation in resistance prediction outcomes, with Mykrobe analysis failing to identify any resistance-conferring mutations in strains JN240_97 and 204_208—a finding inconsistent with TB-Profiler’s predictions and the result of DNA microarray chip method. This discordance underscored the differential sensitivity thresholds of these computational pipelines for detecting *M. tuberculosis* drug resistance markers.

**TABLE 3:** Comparison of drug resistance-associated mutations identified through WGS by TB-Profiler and DNA microarray chip analysis

Sample	RFP mutation sites detected through WGS by TB-Profiler	INH mutation sites detected through WGS by TB-Profiler	RFP mutation sites detected through WGS by mykrobe	INH mutation sites detected through WGS by mykrobe	RFP mutation sites detected by DNA microarray chip analysis	INH mutation sites detected by DNA microarray chip analysis
143_208	*rpoB* p.Asp435Gly (0.99), *rpoB* p.Met434Val (0.99)	*katG* p.Ser315Thr (1.00)	–^*a*^	*katG* p.Ser315Thr	–	–
151_208	*rpoB* p.Ser450Leu (1.00)	–	*rpoB* p.Ser450Leu	*–*	*rpoB* 531 (C->T)	–
177_208	*rpoB* p.Leu452Pro (0.99)	*katG* p.Ser315Thr (1.00)	*rpoB* p.Leu452Pro	*katG* p.Ser315Thr	*rpoB* 533 (T->C)	*katG* 315 (G->C)
205_208	*rpoB* p.Ser450Leu (0.99)	*inhA* c.-777C->T (0.99), *inhA* p.Ser94Ala (0.99)	*rpoB* p.Ser450Leu	*inhA* p.Ser94Ala, *fabG1* (C-15 T)	–	–
JN240_71	*rpoB* p.Ser450Leu (0.96)	–	*rpoB* p.Ser450Leu	*–*	*rpoB* 531 (C->T)	–
JN240_97	*rpoB* p.Leu449Gln (0.10)	–	*–*	*–*	*rpoB* 511 (T->C)	–
107_208	–	*katG* p.Ser315Thr (1.00)	–	*katG* p.Ser315Thr	–	*katG* 315 (G->C)
125_208	–	*katG* p.Ser315Thr (0.92)	–	*katG* p.Ser315Thr	–	–
179_208	–	*katG* c.463delT (0.13)	–	–	–	–
188_208	–	*ahpC* c.-74G->A (1.00)	–	*katG*_W191G	–	–
204_208	–	*ahpC* c.-81C->T (0.99)	*–*	*–*	*rpoB* 531 (C->T)	–

^*a*^ "–" indicates that no drug resistance-associated mutations were detected in the corresponding gene for that strain.

### Genetic distance and clustering analysis

**Fig 3:**
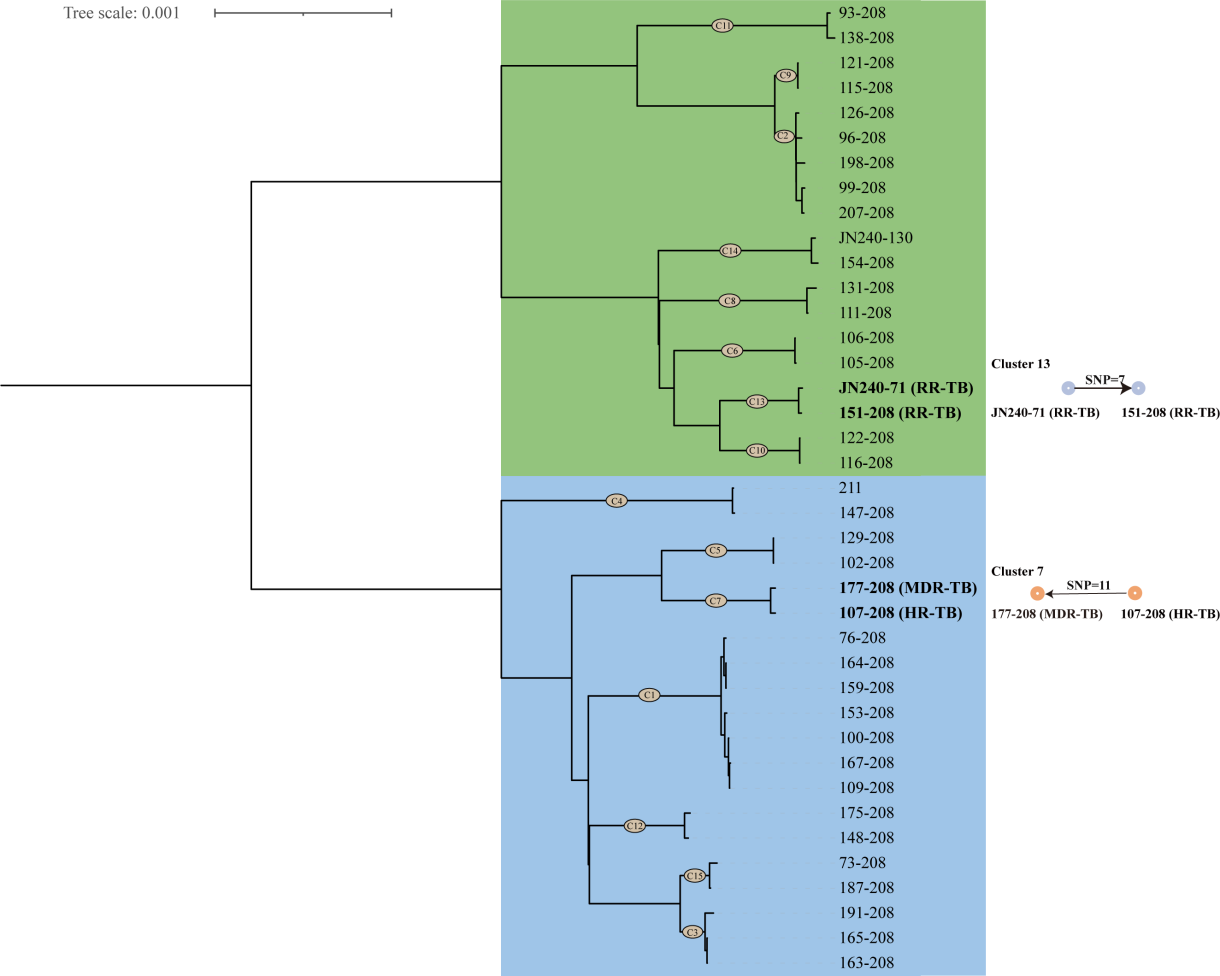
Minimum spanning tree (MST) of clustered isolates based on core genome MLST (cg-MLST) analysis with a 12-SNP clustering threshold. The colors indicate different lineages (green, lineage 4; blue, lineage 2).

WGS analysis of 123 clinical isolates in 2024 revealed distinct transmission clusters through phylogenetic reconstruction using minimum spanning trees (SNP-based) and core genome MLST (≤12 allelic difference threshold). Among 15 identified clusters (39 isolates, 31.7%), cluster sizes varied from two to seven genetically linked cases (Fig. 3). Further transmission analysis, based on the time of diagnosis, revealed two putative recent transmission links. Specifically, strain 177–208 was likely transmitted from strain 107–208, while strain 151–208 potentially originated from strain JN240-71. While the two-case clusters may represent potential household or close-contact transmission, the seven-case cluster suggests possible extended transmission networks, though these patterns do not necessarily confirm specific transmission settings without additional epidemiological data. RFP/INH-resistant strains accounted for 13.3% (2/15) of clusters, indicating recent transmission of pre-resistant genotypes.

**Fig 4:**
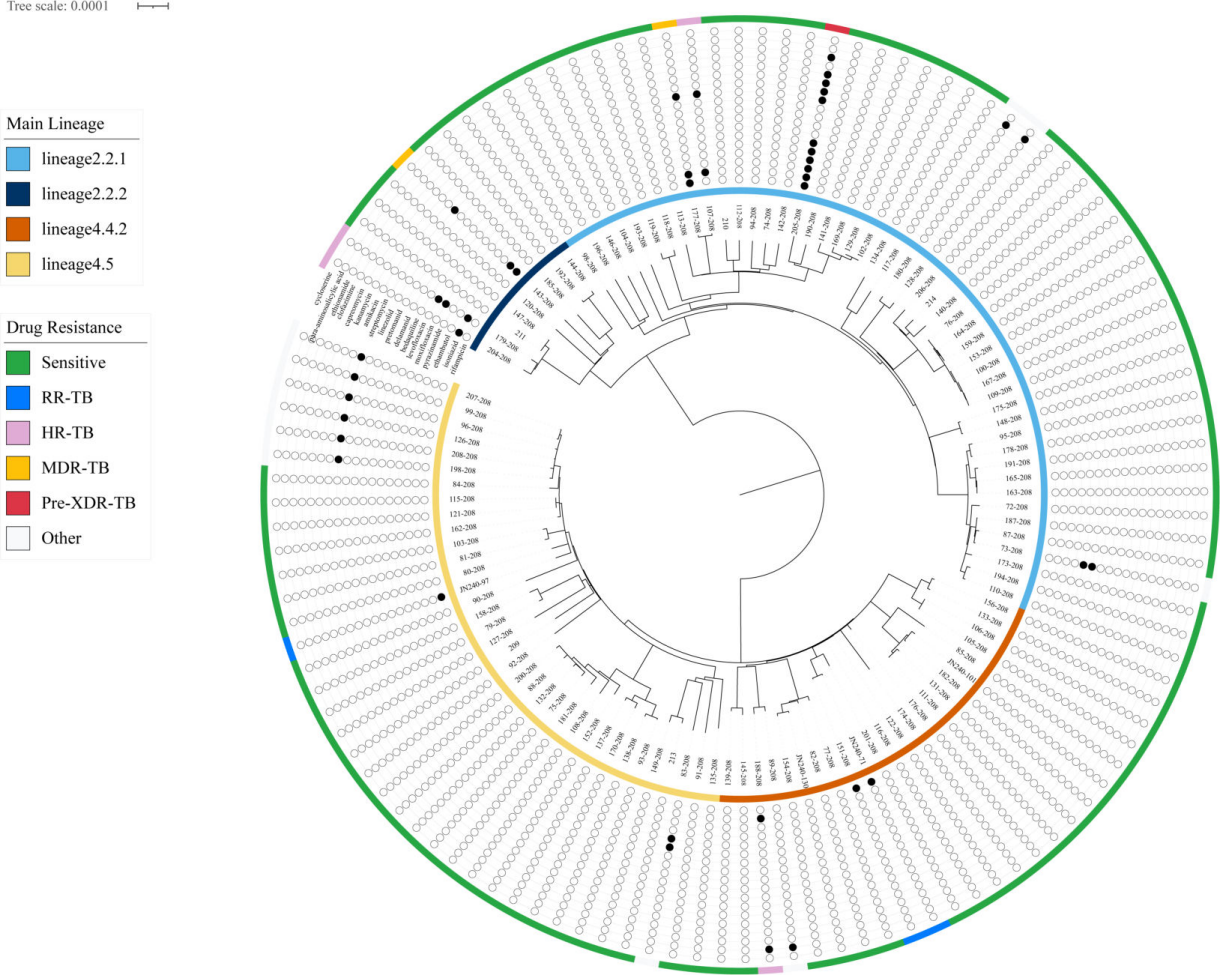
Maximum-likelihood phylogenetic tree of 119 strains based on whole-genome SNPs. The inner ring displays strain lineages (Lineage 2: red; Lineage 4: orange). The outer ring annotates resistance profiles: drug-susceptible (light green), INH-resistant (HR: violet), RFP-resistant (RR: sky blue), multidrug-resistant (MDR: orange), and pre-extensively drug-resistant (pre-XDR: scarlet). Binary antibiotic resistance patterns for 18 drugs are shown as concentric circles (black: resistant; white: susceptible).

TB-Profiler analysis revealed ambiguous phylogenetic classification for five strains. Among these, four isolates (124_208, 125_208, 136_208, and 157_208) exhibited genomic features compatible with both Lineage 2 and Lineage 4. Similarly, strain 123_208 displayed an inconclusive classification pattern, with genetic markers matching characteristics of both sublineages 4.4.2 and 4.5, suggesting possible mixed infection or ancestral recombination. To ensure phylogenetic accuracy, these strains displaying mixed lineage characteristics were omitted from subsequent analysis. Phylogenetic reconstruction was then performed using whole-genome core SNP data from the remaining 118 isolates, with the resulting phylogeny presented in Fig. 4. Among the studied isolates, 58 were identified as Lineage 2 (East Asian branch), with the majority (49 strains) classified as sublineage 2.2.1 and the remaining nine as sublineage 2.2.2. In contrast, 61 isolates clustered into Lineage 4 (Euro-American branch), of which 24 belonged to sublineage 4.4.2 and 36 to sublineage 4.5. The prevalent TB bacteria in the Yi ethnic group predominantly belong to Lineage 2 (21). However, among the isolates collected in this study during 2024, Lineage 2 and Lineage 4 strains were identified at comparable frequencies. *χ* analysis revealed no significant differences in drug resistance between Lineage 2 and Lineage 4 strains for either RR-TB or MDR-TB (both *P* > 0.05).

### Risk factors associated with transmission clusters

**Fig 5:**
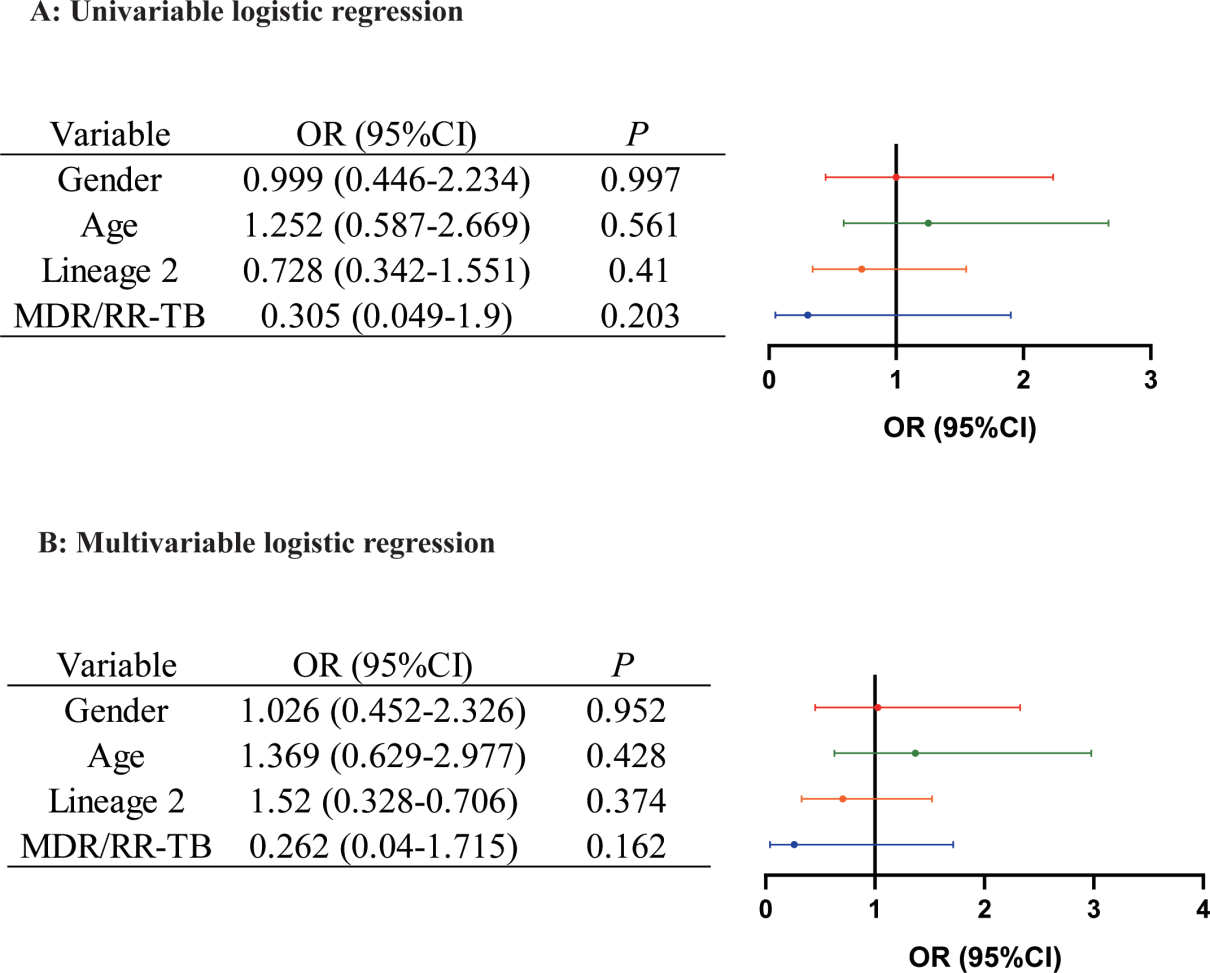
(**A**) Univariable and (**B**) multivariable logistic regression analysis of risk factors for isolates of *M. tuberculosis* transmission clusters. MDR/RR-TB, multidrug-resistant or RR-TB.

In order to delineate the epidemiological features associated with enhanced transmissibility, we conducted a comparative analysis of demographic and clinical characteristics between genomically clustered (indicating recent transmission) and non-clustered isolates (Table 4). Statistical analysis revealed no significant differences in clustering rates among isolates when stratified by lineage, age group, or sex (all *P* > 0.05). Furthermore, our evaluation of clustering patterns by drug resistance profiles demonstrated that, with the notable exception of amikacin-resistant strains (*P* = 0.001), none of the other resistance patterns showed significant associations with bacterial clustering (all *P* > 0.05). Factors demonstrating significant association with disease clustering were incorporated into both univariate and multivariate logistic regression models (Fig. 5). Univariate logistic analysis revealed that neither sex, age, lineage, nor MDR/RR-TB status served as independent risk factors for recent TB transmission. Subsequent multivariate logistic regression modeling indicated comparable clustering probabilities between MDR/RR-TB strains with resistance mutations and drug-susceptible strains (adjusted OR 0.262, 95% CI 0.04–1.715; *P* = 0.162).

**TABLE 4:** Univariate analysis of the risk factor for recent transmission among patients

Factor	Noncluster (*N* = 84)		Cluster (*N* = 39)		*c* ^2^	*P*
	No.	%	No.	%		
Gender						
Male	56	66.7	27	69.2	0.080	0.473
Female	28	33.3	12	30.8		
Age, yr						
3–18	20	23.8	7	17.9	2.608	0.303
19–60	48	57.1	28	71.8		
≥61	16	19.0	4	10.3		
Lineage						
Lineage 2	38	45.2	20	51.3	1.617	0.462
Lineage 4	42	50.0	19	48.7		
Lineage 2; Lineage 4	4	4.8	0	0.0		
RR						
R	3	3.6	2	5.1	0.155	0.514
S	80	95.2	37	94.9		
INH-resistant						
R	6	7.1	2	5.1	1.920	0.213
S	78	92.9	37	94.9		
Moxifloxacin-resistant						
R	3	3.6	1	2.6	0.086	0.622
S	81	96.4	38	97.4		
Streptomycin-resistant						
R	4	4.8	0	0.0	1.920	0.213
S	80	95.2	39	100.0		
Para-aminosalicylic acid-resistant						
R	3	3.6	0	0.0	1.428	0.315
S	81	96.4	39	100.0		
Cycloserine-resistant						
R	5	6.0	0	0.0	2.420	0.143
S	79	94.0	39	100.0		
Amikacin-resistant						
R	1	1.2	7	17.9	12.300	**0.001^*a*^**
S	83	98.8	32	82.1		

^*a*^ Bold values indicates the *P*-value is less than 0.05, highlighting the comparisons where the inter-group differences achieve statistical significance.

## DISCUSSION

RFP and INH are essential first-line anti-TB agents, yet their efficacy is increasingly compromised by the emergence of DR strains. Conventional drug susceptibility testing, though valuable, often lacks the speed required for timely clinical decision-making (22). A critical need exists for rapid molecular assays that can detect *M. tuberculosis* resistance to RFP and INH. DNA microarray technology, developed in the 1990s, offers a promising solution due to its advantages in speed, accuracy, efficiency, and operational simplicity. This platform employs immobilized oligonucleotide probes to detect mutations in specific target genes and has been widely applied for *M. tuberculosis* identification and drug resistance profiling. In an analysis of 4,148 acid-fast *bacilli*-positive sputum samples, Feng et al. (23) demonstrated that microarray-based assays are clinically viable for direct TB diagnosis, showing particularly superior performance in Ziehl-Neelsen smear grade 2+ (ZN 2+) specimens. Similarly, Tang et al. (24) validated the utility of DNA microarray technology for large-scale screening of RFP and INH resistance through detection of mutations in the *rpoB* and *katG* genes, reinforcing its potential as a rapid and accurate diagnostic tool.

An epidemiological study by Han et al. (25) covering DR-TB in Sichuan Province, China, during 2012–2017 reported notably higher drug resistance rates in this region compared to national averages. Located in the Liangshan Yi Autonomous Prefecture, an underdeveloped ethnic minority area of Sichuan Province, China, Meigu County confronts a severe TB epidemic. Contributing factors include limited healthcare infrastructure, persistent economic constraints, and inadequate public attention to infectious disease prevention. Using DNA microarray chip technology, this study profiled drug resistance in *M. tuberculosis* isolated from BALF samples of TB patients at Meigu County Hospital (2022–2024). To investigate associations between various factors and DR strains, *χ* analysis was performed. The analysis revealed a significantly higher risk of INH-resistant *M. tuberculosis* infection in patients aged 18–60 years compared to those in the <18 years and >60 years age groups (*χ*² = 7.034, *P* = 0.022). This age distribution pattern aligns with that observed in MDR-TB patients from Sichuan Province (25). The elevated risk observed in the 18–60 age group, which constitutes the primary workforce, may be attributed to several factors. This demographic often faces substantial economic pressures and life burdens, which can impair their capacity for effective health management. Furthermore, extensive social interactions increase exposure risks, while occupational demands contribute to poorer treatment adherence, thus leading to incomplete or irregular therapy. High population mobility within this group further compounds the issue. Similarly, a study by Qi et al. (26) investigating the epidemiological characteristics and drug resistance profiles of TB patients in northern China (2014–2016) reported that the majority of patients within their cohort were aged 40 to 60 years. In addition, the study by Pan et al. (27) on risk factors for MDR-TB in Dalian, China, between 2013 and 2020 identified the 45–64 years age group as a significant risk factor. Older patients often present with a higher burden of chronic comorbid conditions, such as diabetes and hypertension. These comorbidities can compromise immune function and elevate the risk of drug resistance to anti-TB medications. For instance, diabetes mellitus is a well-known risk factor that not only increases susceptibility to TB but also complicates its treatment (28), which in turn raises the likelihood of the development of drug resistance. Consequently, the middle-aged and elderly group poses a substantial public health risk as a potential reservoir for transmitting DR-TB if treatment management is not intensified.

Notably, the findings of this study further indicated a biphasic trend (initial increase followed by decline) in resistance rates for all three categories: RR-TB, HR-TB, and MDR-TB. Among them, HR-TB resistance peaked at 9.09% (from 6.25%) before declining to 2.25%, demonstrating statistical significance (*χ*² = 6.462, *P =* 0.038). Concurrently, the number of pulmonary TB cases in Meigu County from 2022 to 2024 exhibited an initial decline followed by a resurgence. This pattern suggested a transient improvement in treatment adherence during public health interventions, specifically following the launch of the “Major Infectious Disease Prevention and Control Campaign” in Liangshan Prefecture (where Meigu County is located) on 26 July 2022. This initiative expanded the scope of targeted diseases from HIV/AIDS alone to a comprehensive approach focusing on HIV/AIDS, hepatitis C, TB, and syphilis through integrated control measures. However, the subsequent relapse due to incomplete therapy indicated that non-standardized treatment remained a primary driver of the fluctuating resistance rates observed in the study. This interpretation is further supported by existing documentation of suboptimal treatment adherence among TB patients in Meigu County (11), collectively emphasizing the necessity of enhancing medication supervision during treatment.

Based on WGS analysis of 123 clinical isolates from TB patients in Meigu County in 2024, a total of 11 DR strains were identified, accounting for 8.9% of the samples. Among these, one isolate (0.8%, 1/123) was classified as Pre-XDR-TB, two (1.6%, 2/123) as MDR-TB, three (2.4%, 3/123) as RR-TB, and four (3.3%, 4/123) as HR-TB. The rates of resistance to RFP, INH, and fluoroquinolones observed in this 2024 Meigu cohort were significantly lower than those previously reported for Sichuan Province and its Yi ethnic population (21). It is also noteworthy that low frequencies of resistance were observed for pyrazinamide (0.8%, 1/123) and kanamycin (0.8%, 1/123), while no resistance to ethambutol or delamanid was detected. These findings suggested that under appropriate medical supervision and standardized treatment, first- and second-line anti-TB regimens could achieve favorable treatment outcomes in this population. The results further underscore the importance of maintaining standardized therapeutic protocols and conducting ongoing drug resistance surveillance.

The majority (>95%) of RFP-resistant isolates harbor mutations within the RFP resistance-determining region (*rpoB* codons 507–533) (29). Mutations at codons Ser531, His526, and Asp516 are particularly frequent and strongly associated with resistance. In this study, six mutated codons—531, 511, 516, 526, 533, and 513—were identified in RR isolates. Among these, *rpoB* 531 was the most frequently mutated site (32.3%, 10/31). These mutation sites are consistent with those commonly reported in RR *M. tuberculosis* strains from multiple geographical regions worldwide (30–32). Existing studies have indicated that the frequency of the *rpoB* 511 (CTG→CCG) mutation in Sichuan is higher than that in other provinces of China and other geographical regions worldwide (33). In this study, the mutation frequency of *rpoB* 511 in Meigu County (16.1%, 5/31) was also observed to be relatively high. Additionally, this study identified six RR *M. tuberculosis* isolates from Meigu County that harbored dual mutation patterns, specifically including concurrent mutations at *rpoB* 511 and *rpoB* 516, *rpoB* 511 and *rpoB* 526, and *rpoB* 516 and *rpoB* 526. Such co-occurring mutations have rarely been documented in previous studies. Resistance to INH primarily arises from mutations in genes encoding key metabolic enzymes, notably *katG* (catalase-peroxidase, activating INH), *inhA* (enoyl-acyl carrier protein reductase, the INH target), *KasA*, *ndh*, and *ahpC* (29). Molecular characterization of *katG* and *inhA* mutations facilitates rapid INH resistance diagnosis. Although mutation patterns show geographical variation, specific alterations like *katG* Ser315Thr (often termed *katG* 315) and the *inhA* promoter mutation (C(−15)T, *inhA*-15) predominate globally (34). In this study, mutations associated with INH resistance were also identified at these two specific sites. Detection of these high-frequency mutations is therefore highly indicative of INH resistance.

To our knowledge, this represented the first molecular epidemiological investigation utilizing WGS to characterize MDR and extensively DR *M. tuberculosis* isolates collected from Meigu County in 2024, with a focus on their drug resistance profiles, genetic diversity, and transmission dynamics. Previous studies have established that mutations in the *rpoB* gene serve as the primary mechanism for RFP resistance, with a C->T substitution at codon 531—resulting in the *rpoB* p.Ser450Leu amino acid change—being the most prevalent variant globally (18). In the current investigation, TB-Profiler revealed a low frequency of RFP resistance-conferring mutations (4.9%, 6/123) among the studied isolates. Of these, the *rpoB* p.Ser450Leu mutation represented the most common alteration (50%, 3/6), a result corroborated by Mykrobe. Consistent with these findings, DNA microarray analysis confirmed the presence of the underlying *rpoB* 531 C->T mutation in isolates 151_208 and JN240_71, both of which were independently identified as carrying this mutation by the two bioinformatic tools. Similarly, for strain 177_208, TB-Profiler and Mykrobe each detected a *rpoB* p.Leu452Pro mutation associated with RFP resistance, which was also validated by DNA microarray via the corresponding *rpoB* 533 T->C alteration.

The Ser315Thr substitution in the *katG* gene is a well-established mechanism conferring resistance to INH. Here, TB-Profiler analysis indicated that only 6.5% (8/123) of strains harbored mutations linked to INH resistance. Among these resistant isolates, the *katG* p.Ser315Thr variant was predominant, accounting for 50% (4/8) of cases. Furthermore, in strains 177_208 and 107_208—both of which were flagged by TB-Profiler and Mykrobe as carrying the *katG* p.Ser315Thr mutation—DNA microarray successfully detected the corresponding *katG* 315 G->C nucleotide change, demonstrating concordance across platforms. While Mykrobe and TB-Profiler exhibited high predictive accuracy for identifying resistance to INH and RFP from WGS data (35), inconsistencies in resistance predictions emerged for a subset of isolates in this study. These discrepancies were observed both between the two bioinformatic tools themselves and when compared to results from DNA microarray chip analysis. This observed variability in resistance calling underscored the challenges in genomic prediction.

Fluoroquinolones represent a critical therapeutic option for MDR-TB, with varying resistance levels linked to specific mutations in the quinolone resistance-determining region of the *gyrA* gene. In the present study, *gyrA* mutations were the most prevalent among those conferring fluoroquinolone resistance, with the p.Asp94Gly substitution being the most frequently observed in clinical isolates. This result aligns with findings from Zhang et al. (36) and Willby et al. (37), who also identified *gyrA*, particularly the Asp94Gly site, as a major mutation in fluoroquinolone-resistant strains.

In the genomic sequence analysis of a clinical isolate from this study, the Met306Ile mutation was detected in the *embB* gene—a recognized marker of ethambutol resistance. Mutations at high-confidence sites in *embB*, particularly codon 306, are known to predict approximately 70% of ethambutol resistance cases. The *embB* gene is part of the *embCAB* operon, which encodes arabinosyltransferases essential for the biosynthesis of arabinogalactan and lipoarabinomannan—key structural polymers of the mycobacterial cell wall (38). Consistent with this, Ramazanzadeh et al. (39) confirmed that *embB* 306 mutations are sufficient to confer ethambutol resistance and recommended their detection be incorporated into rapid molecular testing strategies.

A notable observation in our study was the discordance in resistance profiling between the DNA microarray and WGS methods. This could be primarily attributed to their fundamental technological differences. The DNA microarray used in this study was designed to detect a limited spectrum of mutations, specifically targeting the wild-type and mutant variants of three genes associated with RFP and INH resistance: *rpoB*, *katG*, and the *inhA* promoter. In contrast, WGS provides a comprehensive, hypothesis-free survey of the entire genome, enabling the prediction of resistance mutations for a broad panel of anti-TB drugs, including RFP, INH, ethambutol, pyrazinamide, levofloxacin, moxifloxacin, delamanid, clofazimine, and para-aminosalicylic acid. This fundamental difference in scope means that the microarray would inherently miss resistance mechanisms mediated by genes outside its predefined targets. For instance, in strain 204_208, WGS identified an INH resistance-associated mutation in the *ahpC* promoter (*ahpC* c.-81C->T)—a gene not covered by the microarray’s design. Furthermore, WGS, with sufficient depth, possesses superior sensitivity for detecting low-level heteroresistance (40), which may fall below the detection threshold of the microarray. Discordances also arose from the bioinformatic pipelines themselves, as evidenced by strains where Mykrobe failed to identify mutations called by both TB-Profiler and the microarray. These findings underscored the methodological limitations and variable sensitivity of each platform, highlighting the necessity for standardized genomic analysis protocols and the potential benefit of using these methods complementarily for robust drug resistance surveillance.

The genomic clustering rate of clinical *M. tuberculosis* strains serves as an indicator of recent local transmission dynamics. In the present study, among 123 isolates obtained from Meigu County in 2024, 39 strains (31.7%) grouped into 15 genomic clusters, with each cluster defined by ≤12 SNPs, suggesting recent transmission of MDR clones. The clustering rate for RFP-/INH-resistant TB was 13.3%. Previously reported clustering rates of MDR/RR-TB in China varied geographically, ranging from 20.8% to 42.8% across different provinces (12, 14, 41, 42). Regional disparities in clustering rates may be influenced by multiple factors, including socioeconomic and healthcare conditions, population mobility, and contact frequency, as well as the intensity of local TB control measures (43). Owing to their high adaptive fitness associated with DR mutations, Lineage 2 MDR-TB strains have exhibited enhanced transmission capacity in eastern, central, and southern regions of China (44). A previous study conducted in Shenzhen also linked *M. tuberculosis* of Lineage 2 to an elevated risk of MDR/RR-TB transmission (12). In contrast, no significant difference in drug resistance rates was observed between Lineage 2 and Lineage 4 among MDR/RR-TB cases in this study. A significantly higher rate of INH resistance was only detected in Lineage 2 strains compared to Lineage 4 among HR-TB cases. Additionally, this study evaluated the association between various risk factors and the clustering of *M. tuberculosis* strains. The results indicated that the clustering rate was significantly higher among amikacin-resistant strains compared to amikacin-susceptible strains (*χ*² = 12.300, *P* = 0.001). In contrast, patient age, gender, and bacterial lineage showed no significant correlation with strain clustering.

This study has several important limitations pertaining to the sample cohort that should be considered when interpreting the results. The most significant limitation is the discrepant timeframe for the application of the two primary methods. While drug resistance profiling via DNA microarray was performed on a robust set of 378 isolates collected from 2022 to 2024, the subsequent WGS analysis was confined to a subset of 123 isolates from the year 2024 only. Consequently, the comparative analysis between the two methods is based on a temporally restricted sample set, and the WGS-based insights into transmission dynamics and genetic diversity may not fully capture the epidemiological trends present across the entire 3-year period. The smaller sample size available for WGS also reduces the power to detect rare mutations or smaller transmission clusters. Future studies with synchronized, multi-year WGS data are warranted to provide a more complete and representative picture of the evolving *M. tuberculosis* population structure and transmission chains in this region.

In addition to the sample-related constraints noted above, several other limitations should be acknowledged. First, the inference of MDR-TB transmission patterns in Meigu County was contingent upon the representativeness of sampling during the investigation period. However, due to limited laboratory capacity, only isolates from 2024 underwent WGS, resulting in a restricted sample size that may not fully capture transmission trends. Second, the absence of disease onset data, patient occupational information, activity ranges, and records of routine close contacts prevented the determination of epidemiological links among cases. Third, the observed clustering rate might have been influenced if some strains actually formed transmission clusters with isolates outside the sampled population. Lastly, while the DNA microarray chip was evaluated in an epidemiological context in this study, its practical value for rapid RR/MDR-TB diagnosis should be further validated through direct comparison with phenotypic drug susceptibility testing in future investigations.

### Conclusions

This study represented the comprehensive genomic epidemiological investigation of DR *M. tuberculosis* in Meigu County, China, utilizing WGS to elucidate transmission dynamics, resistance patterns, and associated risk factors. Our findings revealed a relatively low overall prevalence of DR pulmonary TB (8.9%) among the 2024 clinical isolates, with distinct resistance profiles and clustering patterns indicative of both ongoing transmission and independent acquisition. The predominance of *rpoB* Ser450Leu and *katG* Ser315Thr mutations aligns with global resistance mechanisms, yet the detection of rare dual mutations underscores unique local genetic diversity. Despite the high-resolution capacity of WGS, discordances between bioinformatic tools (TB-Profiler, Mykrobe) and DNA microarray highlighted persistent methodological challenges in resistance prediction. In contrast to earlier reports on *M. tuberculosis* infections in the Yi population, the lineage distribution among isolates collected for WGS in this 2024-based study differed notably, with Lineage 2 and Lineage 4 strains exhibiting comparable prevalence and no significant disparity. Phylogenetic analysis further identified recent transmission clusters, accounting for 31.7% of the isolates. These clusters were particularly associated with amikacin-resistant strains, suggesting potential nosocomial or community transmission hotspots. These findings underscore the critical need for enhanced genomic surveillance, standardized treatment protocols, and targeted public health interventions to curb the transmission of DR pulmonary TB in underserved high-burden regions like Meigu County.

## SUPPLEMENTAL MATERIAL

Table S1 spectrum.02685-25-s0001.xlsx Genome assembly characteristics of BAL-derived isolates of *M. tuberculosis* following WGS analysis.

ASM does not own the copyrights to Supplemental Material that may be linked to, or accessed through, an article. The authors have granted ASM a non-exclusive, world-wide license to publish the Supplemental Material files. Please contact the corresponding author directly for reuse.
